# Associations of genetic polymorphisms of SAA1 with cerebral infarction

**DOI:** 10.1186/1476-511X-12-130

**Published:** 2013-08-29

**Authors:** Li-Jun Zhang, Bin Yuan, He-Hua Li, Sheng-Bo Tao, Hai-Qing Yan, Li Chang, Jian-Hua Zhao

**Affiliations:** 1 Department of Neurology, First Affiliated Hospital of Xinxiang Medical College, Xinxiang, Henan 453100, P R. China

**Keywords:** Genetic polymorphisms, Serum amyloid A, Cerebral infarction

## Abstract

**Background:**

Serum amyloid A protein (SAA) is both an inflammatory factor and an apolipoprotein. However, the relation between genetic polymorphisms of SAA and cerebral infarction (CI) remains unclear.

**Methods and results:**

The previously reported 4 Single Nucleotide Polymorphisms (rs12218, rs4638289, rs7131332, and rs11603089) of SAA1 gene were genotyped by TaqMan method in a case–control study including 287 cerebral infarction patients and 376 control subjects. We found rs12218 CC genotype and rs7131332 AA genotype were more frequent among CI patients than among controls (9.76% versus 3.19%, P = 0.001; 32.75% versus 24.20%; p = 0.017; respectively). After adjustment of confounding factors such as sex, age, smoking, drinking, hypertension, diabetes, and lipids profile, the difference remained significant in rs12218 (P < 0.01, OR = 2.106, 95% CI: 1.811–7.121).

**Conclusion:**

Genetic polymorphism of SAA1 may be a genetic maker of cerebral infarction in Chinese.

## Introduction

Stroke is the second leading cause of death throughout the world, causing more serious chronic disabilities than any other diseases [1]. Many studies demonstrated that ischemic stroke including cerebral infarction is a multifactor disorder resulting from the interaction between environmental factor and genetics background [2-5].

Serum amyloid A (SAA) is a sensitive acute phase proteins in plasma, also SAA is an apolipoprotein that can replace apolipoprotein A1 (apoA1) as the major apolipoprotein of high-density lipoprotein (HDL), particularly during the acute phase response [6]. Recently, several studies reported the association of SAA1 genetic polymorphism with carotid atherosclerosis [7], lipids levels [8], Uric acid level [9], and peripheral arterial disease [10]. However, the relationship between genetic polymorphisms of SAA and cerebral infarction remains unknown.

In this exploratory study, we investigated the association between genetic polymorphisms of SAA1 and cerebral infarction in a Chinese population.

### Subjects and methods

The present study was approved by the Ethics Committee of the First Affiliated Hospital of Xinxiang Medical College and was conducted according to the standards of the Declaration of Helsinki. Written informed consent was obtained from the participants.

The present study included 287 unrelated adult Chinese patients with acute hemispheric ischemic stroke and 376 symptom free Chinese controls. Cases were selected among patients suffering from atherothrombotic ischemic stroke admitted to the neurology services, within 24 h after onset. Recruitment of the patients was performed consecutively. Stroke was defined as clinical designation for a rapidly developing loss of brain functions that lasted at least 24 h and had no apparent cause other than that of vascular origin. The cerebral infarction was initially diagnosed on the basis of neurological examination and brain computer tomography (CT) scan and then transthoracic echocardiographic examination, Holter study and Transcranial Doppler emboli detection procedure to rule out emboli source. In order to be considered eligible, the patients should meet following criteria: having anterior circulation stroke, no other major illnesses, including autoimmune diseases, neoplasms, coagulopathies, hepatic or renal failure, no known embolic source (aortic arch, cardiac or carotid), no family history of hematological, autoimmune or chronic inflammatory diseases, and no history of myocardial infarction within 3 weeks.

Control subjects were selected randomly from the neurology outpatient clinics who did not have stroke or transient ischemic attack at any time. All exclusion criteria were applied to the controls exactly plus not having ischemic heart disease, carotid stenosis (lumen narrowing) or ulcerated carotid plaque.

### Genotyping

Blood samples were collected from all participants, and genomic DNA was extracted from the peripheral blood leukocytes by DNA extraction Kit (Beijing Bioteke Co. Ltd. China). We selected the tagging single-nucleotide polymorphisms (SNPs) according to the previous studie [9]. Four tagging SNPs (rs12218, rs4638289, rs7131332 and rs11603089) for Chinese Han were genotyped by TaqMan method as described previously [11].

### Biochemical analysis

Serum and plasma collected for measurement was immediately frozen at - 80°C until analysis. We measured the plasma concentration of total cholesterol and HDL cholesterol and the serum concentration of creatinine and uric acid for all the subjects.

### Statistical analyses

Analyses were carried out using SPSS version 17.0 (SPSS, Chicago, IL, USA). The Hardy–Weinberg equilibrium was assessed by chi-square analyses. The differences in the distribution of genotypes between cerebral infarction patients and control subjects were analyzed using the chi-square test. Logistic regression analyses were used to assess the contribution of the major risk factors. Two-tailed p -values, 0.05, were considered significant.

## Results

### Characteristics of study participants

The clinical and metabolic characteristics of the study population are shown in Table 1.

**Table 1:** Characteristics of the participants

**Groups**	**N**	**Age (years)**	**BMI Kg/m**^**2**^	**SBP (mmol/L)**	**DBP (mmol/L)**	**Uric acid (mmol/L)**	**GLU (mmol/L)**	**TG (mmol/L)**	**TC (mmol/L)**	**HDL-C (mmol/L)**	**LDL-C (mmol/L)**
Case group	287	61.1 ± 10.4	23.2 ± 3.6	144.6 ± 22.1	89.2 ± 12.4	302.4 ± 130.3	7.1 ± 3.7	3.14 ± 2.11	5.3 ± 2.5	1.9 ± 1.3	2.82 ± 1.42
Control group	376	62.2 ± 10.5	23.9 ± 3.1	126.1 ± 18.4	78.1 ± 11.9	278.3 ± 81.2	5.3 ± 1.6	1.44 ± 1.21	4.7 ± 1.5	1.7 ± 0.9	2.38 ± 1.11
*P*		0.14	0.33	<0.001	<0.001	<0.001	<0.001	<0.001	<0.001	0.19	0.07

### SAA1 genotype and allele frequencies

All genotyped SNPs were in Hardy-Weinberg equilibrium in the control group (P > 0.05, data not shown). Table 2 shows detailed information for each SNP as well as the allele frequencies.

**Table 2:** Distributions of SAA1 genotypes

**SNPs**	**Allels (1/2)**	**Groups**	**n**	**Genotypes (n,%)**			***P *value**	**MAF**	***P *value**
				**1/1**	**1/2**	**2/2**			
rs11603089	A/G	Control	376	271 (72.07)	98(26.06)	7(1.86)	0.610	0.15	0.364
		Case	287	199(69.34)	80(27.87)	8(2.79)		0.17	
rs4638289	A/T	Control	376	150(39.89)	189(50.27)	45(11.97)	0.102	0.37	0.372
		Case	287	112(39.02)	155(54.01)	20(6.97)		0.34	
rs12218	C/T	Control	376	218(57.98)	146(38.83)	12 (3.19)	0.001	0.23	0.004
		Case	287	145(50.52)	114 (39.72)	28(9.76)		0.30	
rs7131332	A/G	Control	376	91(24.20)	224(59.57)	61(16.22)	0.017	0.46	0.252
		Case	287	94 (32.75)	140 (48.78)	53(18.47)		0.43	

### Association of SAA1 gene polymorphisms and CAD

We found rs12218 CC genotype and C allele were more frequent among cerebral infarction patients than among controls subjects (9.76% versus 3.19%, P = 0.001; 0.30 versus 0.23, P = 0.004; respectively). For rs7131332, we found AA genotype was associated with cerebral infarction (32.75% versus 24.20%, P = 0.017). After adjustment of confounding factors such as sex, age, smoking, alcohol consumption, hypertension, diabetes, and lipids levels, the difference remained significant between the cerebral infarction patients and the control subjects in rs12218 ((P < 0.01, OR = 2.106, 95% CI: 1.811–7.121).

## Discussion

In the present study, we found that rs12218 variation in the SAA1 gene was associated with cerebral infarction in a Chinese population. To the best of our knowledge, this is the first study to investigate the common allelic variants in SAA1 gene and its association with cerebral infarction in Chinese population.

SAA1 encodes one important inflammation factor, SAA, which is also a kind of apolipoprotein [9]. In plasma, SAA is associated with HDL [7,12,13] and, during severe inflammation, can contribute about 80% of its apo-protein composition [14,15]. Therefore, SAA1 is candidates for atherosclerosis and cerebral infarction. Recently, Xie et al. reported that rs12218 polymorphism in SAA1 gene was associated with IMT [8], HDL-C [9], Ankle-brachial index (ABI) [10], and plasma Uric acid levels [11] which was related to cardiovascular and cerebrovascular disease.

In the present study, we performed a case–control study to observe the relationship between SAA1 genetic polymorphism and cerebral infarction. We found rs12218 CC genotype was very common in the cerebral infarction patients than that in the control subjects. After adjustment some confounders, the association remains significant, which indicated that rs12218 CC genotype was an independent risk factor for cerebral infarction.

The mechanisms which may link SAA1 genetic polymorphism to cerebral infarction are largely unknown. According to the previous studies, plasma HDL-C and SAA levels is demonstrated to be associated with SAA polymorphism, which may be a possible mechanism linking SAA1 genetic polymorphism to cerebral infarction which merits further investigation. In addition, the present study was limited by the relatively small sample size. This may have led to weak statistical significance and wide CIs when estimating OR.

In conclusion, the SAA1 genetic polymorphisms were associated with cerebral infarction in a Chinese population.

## Abbreviations

SAA: Serum amyloid A; CI: Cerebral infarction; HDL-C: High-density lipoprotein; LDL-C: Low-density lipoprotein.

## Competing interests

The authors declare that they have no competing interests.

## Authors’ contributions

LJZ and BW carried out the molecular genetic studies and drafted the manuscript. HHL and SBT carried out the genotyping. HQY, LC participated in the design of the study and performed the statistical analysis. JHZ and LJZ conceived of the study, and participated in its design and coordination and helped to draft the manuscript. All authors read and approved the final manuscript.
